# Study of wave propagation in discontinuous and heterogeneous media with the dynamic lattice method

**DOI:** 10.1038/s41598-022-10381-y

**Published:** 2022-04-15

**Authors:** Amir S. Sattari, Zarghaam H. Rizvi, Hendrawan D. B. Aji, Frank Wuttke

**Affiliations:** Geomechanics and Geotechnics Group, University of Kiel, Kiel, Germany

**Keywords:** Civil engineering, Computational science, Scientific data, Applied mathematics

## Abstract

The development of a new dynamic lattice element method (dynamicLEM) as well as its application in the simulation of the propagation of body waves in discontinuous and heterogeneous media is the focus of this research paper. The conventional static lattice models are efficient numerical methods to simulate crack initiation and propagation in cemented geomaterials. The advantages of the LEM and the developed dynamic solution, such as simulation of arbitrary crack initiation and propagation, illustration and simulation of existing inherent material heterogeneity as well as stress redistribution upon crack opening, opens a new engineering field and tool for material analysis. To realize the time dependency of the dynamic LEM, the equation of motion of forced vibration is solved while using the Newmark-$\beta$ method and implementing the non-linear Newton–Raphson Jacobian method. The method validation is done according to the results of a boundary element method (BEM) in the plane P-SV-wave propagation within a plane strain domain. Further tests comparing the generated wave types, simulation and study of crack discontinuities as well as inherent heterogeneities in the geomaterials are conducted to illustrate the accurate applicability of the new dynamic lattice method. The results indicate that with increasing heterogeneity within the material, the wave field becomes significantly scattered and further analysis of wave fields according to the wavelength/heterogeneity ratio become indispensable. Therefore, in a heterogeneous medium, the application of continuum methods in relation to structural health monitoring should be precisely investigated and improved. The developed dynamic lattice element method is an ideal simulation tool to consider particle scale irregularities, crack distributions and inherent material heterogeneities and can be easily implemented in various engineering applications.

## Introduction

In civil engineering and material science, continuum-based methods such as boundary element, finite element, finite difference and volume element methods are the most common practices to simulate the dynamic wave propagation through solid bodies. These methods have been developed under the consideration of a wide range of boundary conditions from the 70’s until present day. However, the simulation of structural vibrations or wave propagation in solids under the consideration of damaged or heterogeneous structures cannot easily be performed using the continuum-based methods. In particular, the consideration of local damages or a stochastically distributed heterogeneity is difficult to realize. The structural health monitoring methods are widely considered and implemented in engineering applications to ensure the usability as well as the state analysis of structures, and finally the failure of structures^1^. The accurate identification of the structural state requires consideration of all disturbances within the structure, such as small damages or material heterogeneity. In general, the dynamical methods differ in structural dynamics based on the analysis of waves to determine existing damages and wave-based methods to identify the location of the discontinuities^2^, often with high frequency waves to detect the damages^3,4^. So far during the past decades, mainly continuum-based approaches were developed to detect the damages in the structures^5^. Finite element analysis is implemented to analyse and detect cracks in a cantilever beam^6^. Advanced wavelet-based neural networks are considered in the detection of damages in beam-like structures^7^. Similarly, a numerical scheme is proposed for the detection of multiple cracks in three dimensional (3D) structures^8^. Within the scope of heterogeneous elasticity, a dispersion equation is derived for a heterogeneous layer over an inhomogeneous half-space, in which the propagation of Love waves are influenced by inhomogeneity parameters^9^. Likewise, the dynamic behavior of multi-layer heterogeneous composite magneto-elastic structures for surface wave scattering is investigated, where a finite difference technique is derived to obtain the group and phase velocities^10^. In another study, it is found that because the medium tends to be elastic as the viscoelastic parameter decreases, the propagation of torsional waves faced considerable hindrance in spreading further^11^.

Besides the continuum-based methods, the development of discrete methods has become very popular to model micro- and meso-scaled problems, mostly to understand the phenomenon of material behaviour. Discrete methods, such as cohesive Discrete Element Method (CDEM), are used, e.g. to simulate the fracking in cemented geomaterials^12,13^. The standard Discrete Element Method (DEM) on the other hand, as an inherent Lagrange based method, is considered for the simulation of large particle movement in non-cohesive granular materials^14^. Application of CDEM is also expanded in different scientific fields, such as simulation of crack propagation in a vitreous biopolymer material^15^. Coupled FED-DEM methods are developed to tackle the computational costs that are associated with discrete methods and are able to simulate fracking while considering the irregularities that exist in geomaterials^16^. However, mainly due to the great numerical costs, the discrete simulation methods were barley considered until now to simulate a full dynamic or wave field problem in engineering applications. To decrease the computational costs and lower the problems of complexity that exist for analysing discrete structures, the so-called lattice element method (LEM) has been developed. The initial idea was to analyse the crack propagation in solid and heterogeneous materials, like concrete^17^.

The LEM can be considered as a hybrid method, where the generated domain can be either seen as a continuum or as packed discrete particles^18^. The lattice elements are able to simulate the frack initiation and propagation in solids and cemented geomaterials, where small particle movements or displacements are expected^19–22^. One of the main advantages of the LEM related to other numerical methods is its ability to simulate the stress redistribution and concentration upon the cracking process. The domain can be represented in the simplest way by a series of springs^19^, more complex than Euler Bernoulli or Timoshenko beams^23^. The generalized beam lattice model, in which mechanical bond-aggregate interface properties are implemented in a lattice element, is also used to simulate the crack growth in concrete^24^. The Rigid-body-spring networks are also similar to lattice elements, representing the domain with a series of spring elements. In these methods, between each unit cell, three spring elements representing a resistance against axial, transverse and rotation displacements are considered^25–27^. The initial step in application of the lattice model is to grant the mesh-independence outcome of the simulations. In this regard, the embedded strong discontinuity is introduced into lattice elements, resulting in mesh-independent computations of failure response^23^. In another approach, the correlation between single element and continuum properties is achieved using the strain energy theory, where a strain energy stored in a unit cell is equal to the strain energy stored in a continuum^28,29^. The crack simulation in lattice elements is performed by removing an element reaching its strength threshold (brittle failure) or decreasing the stiffness according to a bi-linear softening scheme. The failure of elements is defined based on the Mohr–Coulomb failure model with a tension cut off^24,25^ or based on mode I and II fracture mechanisms, where a critical stress intensity factor is used to calculate the released strain energy rate^19,30,31^. In all the aforementioned developments, the focus was on the study of fracture propagation in materials.

Over the last few years, the application of lattice elements has been extended to simulate the coupled thermo-hydro-mechanical processes. The heat transfer and change of effective thermal conductivities in cemented geomaterials^32^, in unsaturated granular geocomposites^33^ or modified geomaterials^34^ have all been investigated. The hydro-mechanical dual lattice models are developed to simulate the flow and permeability changes in geomaterials^35,36^. The dual lattice model was also implemented to investigate the fluid-driven percolation and developed flow paths in salt and clay stone samples under anisotropic confining stresses^18^. The initial approach to extend the common lattice element method into the field of dynamics for use of wave field simulation in heterogeneous materials has already been developed^37^. Here, the dynamic LEM is used to simulate wave fields while considering a progressive fracking process in brittle and quasi-brittle material under dynamic loading. Additionally, the dynamic LEM is applied to solve the problem of mechanical waves in rock mass or cemented granular material under dynamic loadings^38^. The simulation of crack propagation under dynamical forces by an embedded strong discontinuity approach is also implemented^39^. The simulation results of the dynamicLEM are used to train the artificial neural networks (ANN) model to detect the location of the discontinuities^40^, thus reducing the computational costs.

This research paper extends the application of the lattice element method in dynamic structural analysis to tackle the effect of discontinuities and inherent material heterogeneities on wave fields. The inherent mesh irregularity of the lattice element and stochastic fracking process makes the model suitable for simulation of discontinuities under expected small deformations. Initially, the implemented mathematical methodology is explained, where Newton’s second law within the equation of motion is solved in the time domain to determine the accelerations, velocities and displacements of generated nodes. Afterwards, validation benchmarks are presented and the results of the dynamic lattice for a 2D domain is compared to the boundary element method (BEM) solution. Within that benchmark, the dynamic structural behaviours are compared in the frequency domain to demonstrate the accuracy of the method. To show the influence of discontinuities, a series of full wave field simulations are performed and analysed. The visualization of the wave types within propagating wave field, the wave field scattering and dispersion at heterogeneities, as well as the wave field shadows around discontinuities illustrate the excellent ability of the new dynamic method and its future applications in structural dynamics and structural health monitoring.

## Methodology and mathematical framework

### Domain discretization and discontinuity

**Figure 1:**
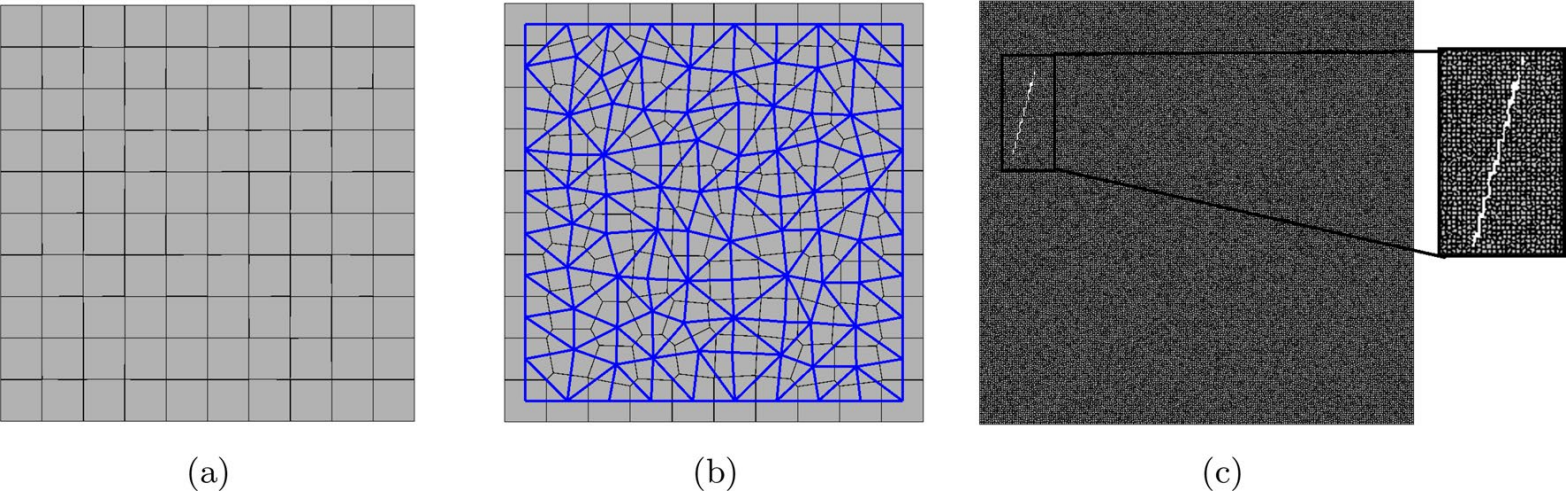
Discretization of a 2D domain: (**a**) generated Voronoi cells, when $\alpha _f=0.01$, (**b**) generated lattice elements using Delaunay Triangulation (blue lines), when $\alpha _f=0.5$, and (**c**) generated random crack, when $\alpha _f=0.5$ and mesh size is 200 by 200.

The general discretization of a domain is performed using the vectorizable random lattice^41^. In the implemented discretization method, the generated irregularity of a mesh is controlled using the defined randomness factor $(\alpha _f)$, where $\alpha _f=1$ is the maximum irregularity and $\alpha _f=0$ generates a regular mesh. After the generation of the nodes, which can all act generally as crack nuclei, the Voronoi Tessellation and Delaunay Triangulation are considered to mesh the domain by generating polygonal cells and their lattice connectivities (Fig. 1a,b). Within the lattice model, these elements represent the domain and carry the mechanical loads^32^. The location, orientation and length of the generated discontinuities are in a stochastic manner as shown in Fig. 1c, in which the nodes that have been intersected by the discontinuity have been removed from the stiffness matrix.

**Figure 2:**
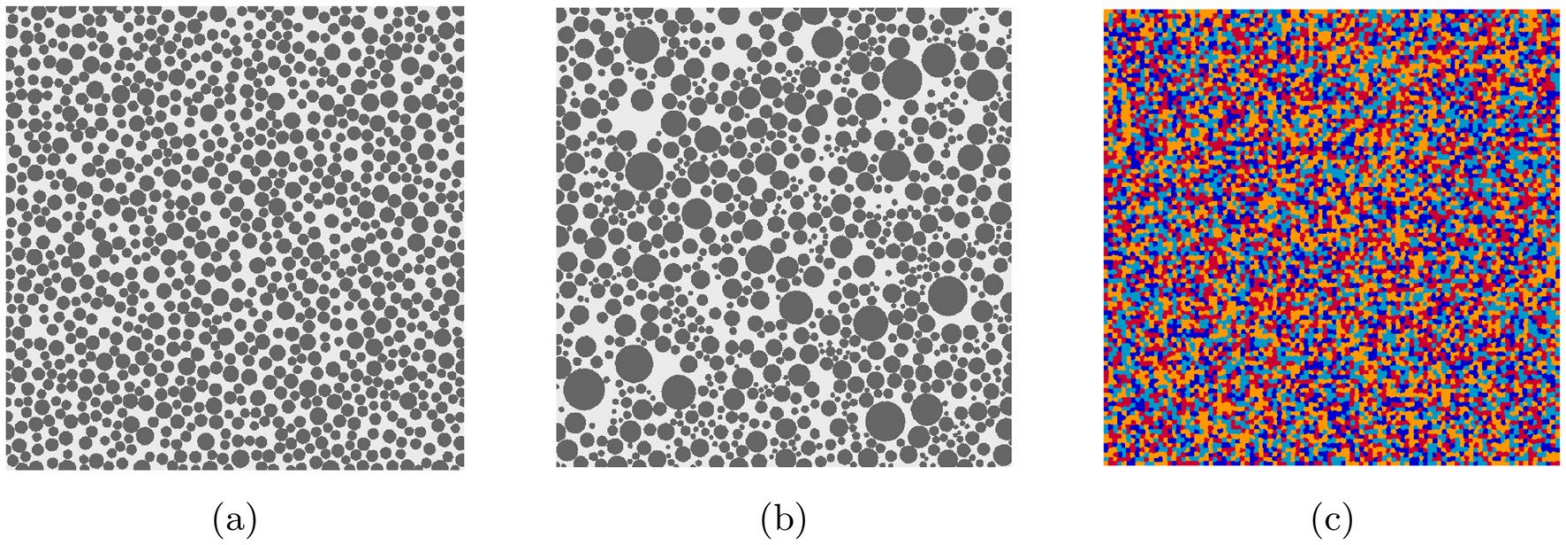
Generated heterogeneous granular domain: (**a**) uniform aggregate-bond distribution, $D=2\sim 4$ mm, (**b**) non-uniform aggregate-bond distribution, $D=1\sim 10$ mm, and (**c**) arbitrarily distributed heterogeneity, when $\alpha _f=0.5$ and mesh size is 600 by 600.

One of the advantages of considering the lattice model is its simplicity of generating a heterogeneous granular domain. Therefore, this numerical method can easily be used in crack simulations in e.g. concrete material, where granular particles are cemented with each other^42,43^. In these models, granular particles, cement (bond material) and interfaces are defined with different mechanical properties. Figure 2 illustrates two different granular packing layouts with uniform and non-uniform granular distributions. The arbitrarily distributed heterogeneity with four different minerals is shown in Fig. 2c. To use the dynamic LEM in engineering applications such as structural health monitoring, the visualisation and quantification of wave fields in heterogeneous materials is of great importance.

### Composition of the static lattice method

**Figure 3:**
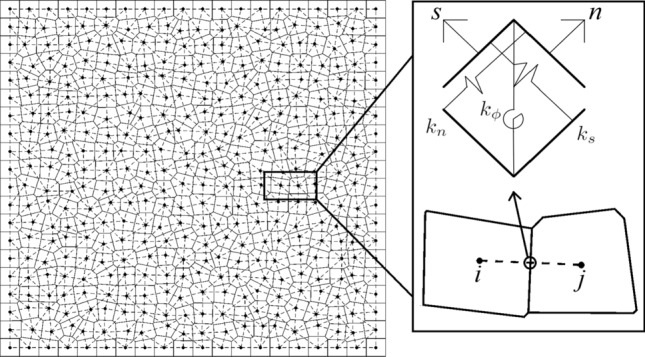
The generated Voronoi cells and the defined spring elements.

Similar to the Rigid-Body-Spring Network methods, the resistance to axial, transverse and rotational displacements are modeled using three spring elements^44^. Implementing the spring elements eliminates the simulation difficulties that arose from short beam elements, such as a small beam aspect ratio. Each generated nuclei has three degrees of freedom and Fig. 3 illustrates the generated Voronoi cells and the defined spring elements among them.

1 $$\begin{aligned} k_s=G\frac{A}{l}, \qquad k_n=E\frac{A}{l}, \qquad k_\phi =k_n\frac{l^2}{12}, \end{aligned}$$

where *A* is the cross-section area ($A=t\times h$), h is the cross-section length of the elements, *t* is the thickness of the domain, *l* is the length of elements or Euclidean distance between nuclei, $k_s$ is the transverse stiffness, $k_n$ is the axial stiffness, $k_\phi$ is the rotational stiffness, *G* is the shear modulus and *E* is the Young’s modulus. While assuming the linear elasticity, according to Hooke’s law,

2 $$\begin{aligned} \varvec{f_g}=\varvec{K_g}\varvec{u_g}, \qquad \varvec{K_g}=\varvec{T}^T\varvec{K_l}\varvec{T}, \end{aligned}$$

where $\varvec{T}$ is the transformation matrix, $\varvec{K_l}$ is the stiffness matrix in local coordinates, $\varvec{f_g}$ is the vector of forces, $\varvec{K_g}$ is the stiffness matrix and $\varvec{u_g}$ is the vector of displacements in the global Cartesian coordinate.

The regularisation of a lattice model is carried out according to the strain energy theory and energy balance, where the stored energy in a continuum ($U_\mathbb {R}$) is considered to be equal to the stored strain energy of a unit cell ($U_{cell}$)^28^. For a spring element, the strain energy stored in a unit cell is determined according to the total number of bond elements ($N_b$), the elements response forces ($f_b$) and the corresponding displacements ($u_b$). In a continuum, the stored energy is calculated with the applied stresses ($\sigma _{\mathbb {R}}$) and strains ($\varepsilon _{\mathbb {R}}$) throughout its volume ($V_{\mathbb {R}}$).

3 $$\begin{aligned} U_\text {cell}=U_\mathbb {R}, \qquad U_\text {cell}=\frac{1}{2}\sum _{b=1}^{b=N_b} f_b \, u_b, \qquad U_\mathbb {R}= \frac{1}{2} \int _{V_{\mathbb {R}}} {\sigma _{\mathbb {R}} \, \varepsilon _{\mathbb {R}} \,dV} \end{aligned}$$

For a unit cell, the stored strain energy is calculated based on the length of the elements (*l*), first stiffness coefficient ($R^{\prime }$) and orientation of the unit normal vector ($n_{i,j,k,m}$).

4 $$\begin{aligned} U_\text {cell}=\frac{1}{2}\sum _{b=1}^{b=N_b} l_b^2 \, \left( R^\prime \, n_i \, n_j \, n_k \, n_m \, \varepsilon _{ij} \, \varepsilon _{km} \right) _b \end{aligned}$$

For a continuum, the stored strain energy is calculated by

5 $$\begin{aligned} U_\mathbb {R} = \frac{1}{2} \varepsilon _{\mathbb {R}} \, C_{\mathbb {R}} \, \varepsilon _{\mathbb {R}}, \end{aligned}$$

where $C_{\mathbb {R}}$ is a continuum stiffness matrix. Finally, a correlation between continuum properties such as Young’s Modulus ($E_{\mathbb {R}}$), Shear Modulus ($G_{\mathbb {R}}$) and Bulk Modulus ($K_{\mathbb {R}}$), with single lattice properties (*E*, *G*, *K*), is driven based on the continuum stress condition and with the linear elastic theory. According to this correlation, the desired Poisson’s ratio ($\nu$) of a continuum will also be achieved. The irregularity of the generated mesh ($\alpha _f$) has an influence on the correlated ratios ($\alpha _1,\alpha _2,\alpha _3$).

6 $$\begin{aligned} \frac{E_{\mathbb {R}}}{E}=\alpha _1, \qquad \frac{G_{\mathbb {R}}}{G}=\alpha _2, \qquad \frac{K_{\mathbb {R}}}{K}=\alpha _3 \qquad \end{aligned}$$

**Figure 4:**
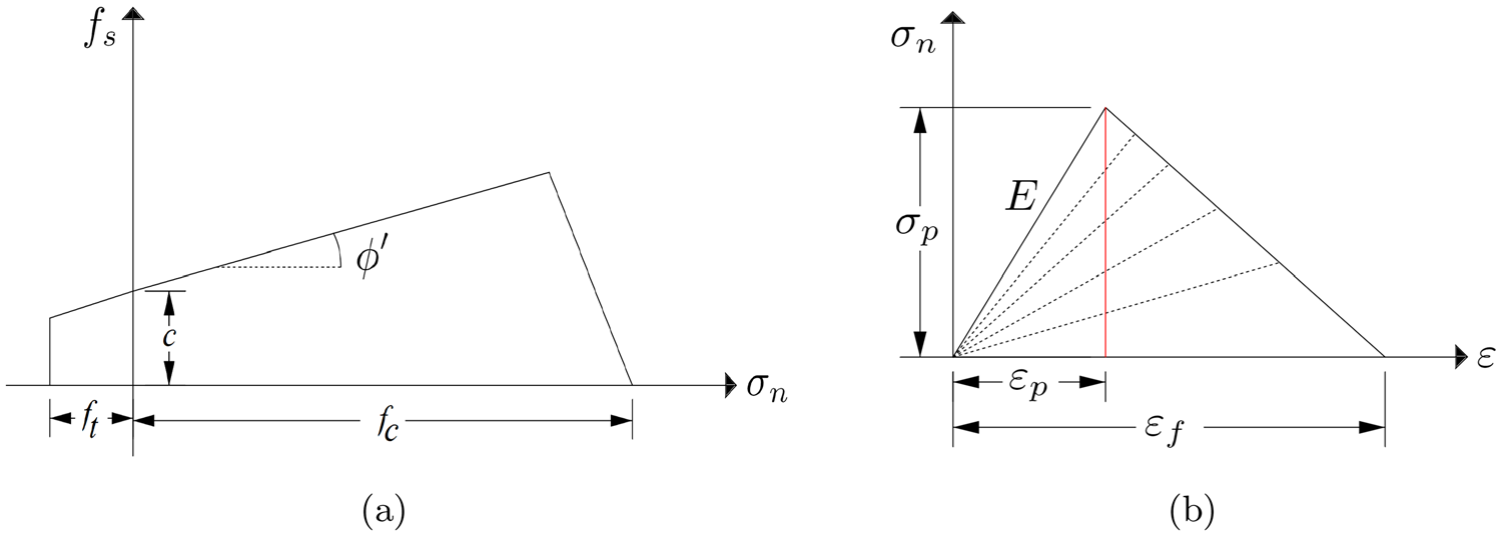
Fracture mechanism: (**a**) Mohr–Coulomb failure model with tension cut, and (**b**) implemented bi-linear softening scheme.

Under the assumption of linear elastic fracture mechanics (LEFM) and while considering the Mode I and II fracture mechanism within the model, the crack initiation and propagation can be simulated. The implemented failure criteria here is based on the Mohr–Coulomb failure model with tension cut off^45,46^. Figure 4a depicts the failure criteria and defined cohesion (*c*) and tensile strength ($f_t$). The elements failure under compression forces (particle crushing) can also be simulated to grant crack propagation.

7 $$\begin{aligned} f_t=\frac{\sigma _n}{A}-\alpha ^{\prime }\frac{12\times max(|M_i|,|M_j|)}{th^2}, \qquad f_s=\sigma _n \cdot \tan (\phi ^{\prime }) + c, \end{aligned}$$

where $\sigma _n$ is the axial stress, $\alpha ^{\prime }$ is a scaling parameter equal to 0.005^47^, $M_{i,j}$ are the moments in node *i* and *j*, *h* is the cross-section length of the elements, $\phi ^{\prime }$ is the friction angle and $f_s$ is the shear strength. Modeling a quasi-brittle material, such as concrete or rock, requires implementation of a softening scheme^47^. Figure 4b illustrates the implemented bi-linear softening scheme, where based on the softening ratio (*SR*), the stiffness of the element is reduced.

8 $$\begin{aligned} E=\frac{\sigma _p}{\varepsilon _f-\varepsilon _p}\left( \frac{\varepsilon _f}{\varepsilon }-1\right) , \qquad SR=\frac{\varepsilon _f}{\varepsilon _p}, \end{aligned}$$

where $\sigma _p$ is the peak stress, $\varepsilon _p$ is the peak strain, $\varepsilon _f$ is the failure strain and $\varepsilon$ is the elements strain. When $SR=1$, a brittle failure is simulated and the element stiffness is deducted to zero (removed). The provided results in this study in “Simulation of wave fields in cracked and heterogeneous materials” and “Validation of the dynamic lattice element method” are solved using the dynamic lattice model, and the static lattice solution provided here is for general understanding of the composition of the lattice model and the extension of the model to the dynamic approach. The simulation of crack initiation and propagation is not within the scope of this study.

### Implementation of the dynamic lattice method

In the dynamic lattice method the equation of motion of forced vibration is solved, in which the accelerations ($\varvec{\ddot{u}}$), velocities ($\varvec{\dot{u}}$) and displacements ($\varvec{u}$) of nodes in each time step (*t*) are calculated according to the applied external load. The mass matrix ($\varvec{M}$) is assembled according to the consistent mass matrix (CMM) by lumping the mass at the nodes. The global stiffness matrix ($\varvec{K_g}$) is assembled as previously shown in Eq. (2) and Eq. (1). The contact forces in the dynamic lattice method in contrast to discrete element models is equal to zero, as no contact search between the elements is required. This reduces the computational costs of the dynamic lattice models. The damping matrix ($\varvec{C}$) is also specified, although the contact damping is neglected here.

9 $$\begin{aligned} \varvec{M}\varvec{\ddot{u}}(t)+\varvec{C}\varvec{\dot{u}}(t)+\varvec{K_g}\varvec{u}(t)=\varvec{f_{ext}}(t), \qquad t_{(i+1)}=t_{(i)}+\Delta t, \end{aligned}$$

where $\varvec{f_{ext}}$ is a vector of external dynamic loads and $\Delta t$ is the time step. Different explicit and implicit methods can be used to solve the equation of motion, such as the central difference method, implicit linear acceleration method and Newmark-$\beta$ method with incremental formulation. In this paper, the equation of motion of forced vibration is solved using Newmark-$\beta$ method with incremental formulation as given below.

10 $$\begin{aligned} \delta \varvec{\ddot{u}}= & {} \frac{1}{\beta \Delta t^2}\delta \varvec{u}-\frac{1}{\beta \Delta t}\varvec{\dot{u}}-\frac{1}{2\beta }\varvec{\ddot{u}}, \nonumber \\ \delta \varvec{\dot{u}}= & {} \frac{\gamma }{\beta \Delta t}\delta \varvec{u}-\frac{\gamma }{\beta }\varvec{\dot{u}}+\Delta t (1-\frac{\gamma }{2\beta })\varvec{\ddot{u}}, \end{aligned}$$

11 $$\begin{aligned} \delta \varvec{\ddot{u}}= & {} \varvec{\ddot{u}}(t_{i+1})-\varvec{\ddot{u}}(t_{i}), \nonumber \\ \delta \varvec{\dot{u}}= & {} \varvec{\dot{u}}(t_{i+1})-\varvec{\dot{u}}(t_{i}), \qquad \delta \varvec{u}=\varvec{u}(t_{i+1})-\varvec{u}(t_{i}), \end{aligned}$$

where $\gamma$, $\beta$ are the Newmark-$\beta$ parameters and $\delta \varvec{u}$, $\delta \varvec{\dot{u}}$, $\delta \varvec{\ddot{u}}$ are the increments of displacement, velocity and acceleration, respectively. The incremental formulation of Newton’s second law is given as,

12 $$\begin{aligned} \varvec{M}\delta \varvec{\ddot{u}}+\varvec{C}\delta \varvec{\dot{u}}+\varvec{K_g}\delta \varvec{u}=\varvec{\delta }{f_{ext}}. \end{aligned}$$

The incremental formulation of the equation of motion can be solved under the assumption of different $\gamma$ and $\beta$ parameters. When $\beta =\frac{1}{4}$ and $\gamma =\frac{1}{2}$, the Newmark method is unconditionally stable and implicit. If $\beta =\frac{1}{6}$ and $\gamma =\frac{1}{2}$, the Newmark method is similar to the linear acceleration method. Under the assumption of $\beta =0$ and $\gamma =\frac{1}{2}$ the Newmark method is identical to the central difference method^48^. The Newton-Raphson Jacobian is implemented here to solve the system of nonlinear equations with multiple variables.

13 $$\begin{aligned} \delta \varvec{u}_{n+1}= & {} \delta \varvec{u}_n-\varvec{J}^{-1}(\delta \varvec{u}_n)\varvec{F}(\delta \varvec{u}_n), \end{aligned}$$

14 $$\begin{aligned} \varvec{F}(\delta \varvec{u}_n)= & {} \varvec{M}\delta \varvec{\ddot{u}}+\varvec{C}\delta \varvec{\dot{u}}+\varvec{K_g}\delta \varvec{u}-\varvec{\delta }{f_{ext}}, \end{aligned}$$

where *n* is the numerical iteration number and $\varvec{J}^{-1}$ is the inverse of the Jacobian matrix, which is equal to the derivative of the $\varvec{F}(\delta \varvec{u}_n)$ with respect to each unknown node displacement. The convergence of the dynamic lattice method in the time domain depends on the wavelength and the length of the lattice elements. The higher frequencies lead to smaller wavelengths, which require smaller elements sizes and increases the computational costs. The developed dynamic lattice method also considers the Sommerfeld radiation conditions for infinite domains by use of Perfectly Matched Layer (PML)^49^ to absorb the waves’ energy at the boundaries and avoid boundary reflections, if it is required.

## Validation of the dynamic lattice element method

The validation of the dynamic lattice method is performed by a comparison of the results obtained from the presented dynamic lattice method with the solution of the Boundary Element Method (BEM) for an elastodynamic problem. The benchmark considered here is a plane P-SV-wave propagation within a plane strain domain. The analytical solution for the one-dimensional shear-wave propagation problem can be easily obtained^50^. In the presented example, the domain is homogeneous.

The BEM is solved according to the boundary integral equation method (BIEM) and with use of a collocation procedure, whereas it can be written as^50^:

15 $$\begin{aligned} c_{lk}u_{k}(x,\omega ) = \int _{\Gamma _{}} U_{lk}^{*}(x,\xi ,\omega )~t_{k}^{}(\xi ,\omega ) d\Gamma -\int _{\Gamma _{}} T_{lk}^{*{}}(x,\xi ,\omega )~u_{k}^{}(\xi ,\omega ) d\Gamma . \end{aligned}$$

Here, $\omega$ is the circular frequency; $x, \xi$ are the coordinates of the source and receiver; $U^{*}, T^{*}$ are the elastodynamic fundamental solutions for displacement and traction, respectively; *t* is the traction acting on the domain boundary $\Gamma$; and *c* is the jump-term which depends on the geometry of the source point.

The fundamental solutions for time-harmonic elasticity are as follows:

16 $$\begin{aligned} U_{lk}^{*}(x,\xi ,\omega )= & {} \frac{1}{2\pi \rho C^2_{2}} \left[ \Psi \delta _{lk}-\chi r_{,l}r_{,k}\right] ; \end{aligned}$$

17 $$\begin{aligned} T_{lk}^{*}(x,\xi ,\omega )= & {} \frac{1}{\alpha \pi }\left[ \left( \frac{d\psi }{dr}-\frac{1}{r}\chi \right) \left( \delta _{lk}\frac{\partial r}{\partial n}+r_{,k}n_l\right) -\frac{2}{r}\chi \left( n_kr_l-2r_{,l}r_{,k}\frac{\partial r}{\partial n}\right) -\frac{2dx}{dr}r_{,l} r_{,k}\frac{\partial r}{\partial n}\right. \nonumber \\&+\left. \left( \frac{C^2_{2}}{C^2_{1}}-2\right) \left( \frac{d\psi }{dr}-\frac{dx}{dr}-\frac{\alpha }{2r}\chi \right) r_{,l}n_{k}\right] , \end{aligned}$$

where

$$\begin{aligned} \Psi =&K_0(k_2~r)+\frac{1}{k_2~r}\left[ K_1(k_2~r)-\frac{C_2}{C_1}K_1(k_1~r)\right] ;\\ \chi =&K_2(k_2~r)-\frac{C_2^2}{C_1^2}K_2(k_1~r). \end{aligned}$$

The notations $C_1$ and $C_2$ are the longitudinal and shear wave velocities, respectively; $K_n$ is the Bessel function of the second kind and *n*^th^ order; $k_1=i(\omega /C_1)$, $k_2=i(\omega /C_2)$ with $i=\sqrt{-1}$; and the subscripts *l* and *k* are the direction of the source load and the receiver response, respectively. Further description of the classical formulation can be found in^50^. After discretization, Eq. (15) is rewritten in matrix notation as:

18 $$\begin{aligned} \mathbf {H}^{}\mathbf {u}^{}=\mathbf {G}^{}\mathbf {t}^{}, \end{aligned}$$

where $\mathbf {H}^{}$ and $\mathbf {G}^{}$ are the influence matrices; and $\mathbf {u}^{}$, $\mathbf {t}^{}$ are the vectors of nodal displacement and traction, respectively. Eq. (18) is solved by: (1) assembling the influence matrices components corresponding to the unknown nodal values as matrix $\mathbf {A}$ in the left hand side; and (2) multiplying the prescribed boundary condition values with their corresponding influence matrix components and summing them in vector $\mathbf {f}$ in the right-hand side, which is given as:

19 $$\begin{aligned} \mathbf {Ax}=\mathbf {f}. \end{aligned}$$

**Figure 5:**
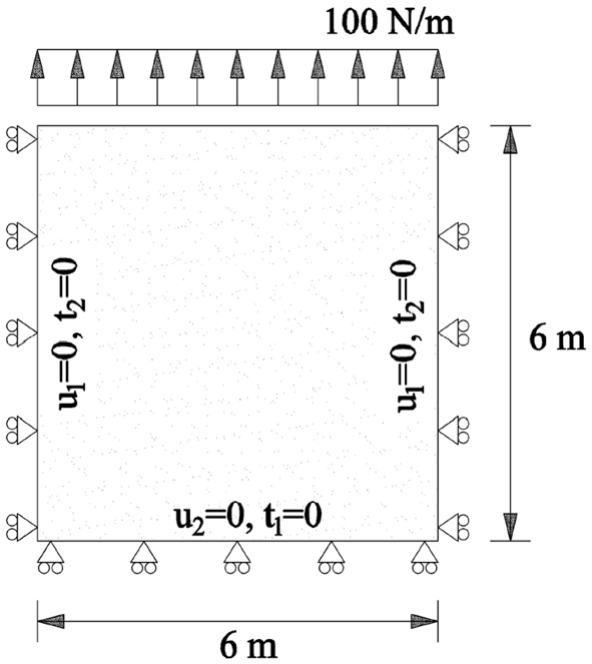
The considered plane strain boundary condition for validation of the dynamic lattice model.

Here, $\mathbf {x}$ is a vector containing the unknown values. The geometry of the benchmark problem is a square with dimensions of 6x6 m (Fig. 5). The material properties are as follows: shear modulus $G=10^{6}$ N/m$^{2}$, density $\rho$=100 kg/m$^{2}$, damping ratio 5 %, and Poisson’s ratio $\nu$=0.25. The prescribed boundary conditions are defined as: (1) zero perpendicular displacement on bottom boundary, (2) zero transverse displacement on the side boundaries, and (3) applied uniform traction of 100 N/m on top boundary. The BEM solution is obtained using quadratic line elements. An analytical solution of the resonance frequencies is given for discrete frequencies as:

20 $$\begin{aligned} \omega _n=(2n+1) \frac{\pi C_1}{2L}, \end{aligned}$$

where *L* is the travel distance, $C_1$ is the P wave velocity and $n=0,1,2~\ldots$, which yields $\omega _n\approx$ 45.40, 136.20, 227.00, 317.81 ...rad/s^50^.

**Figure 6:**
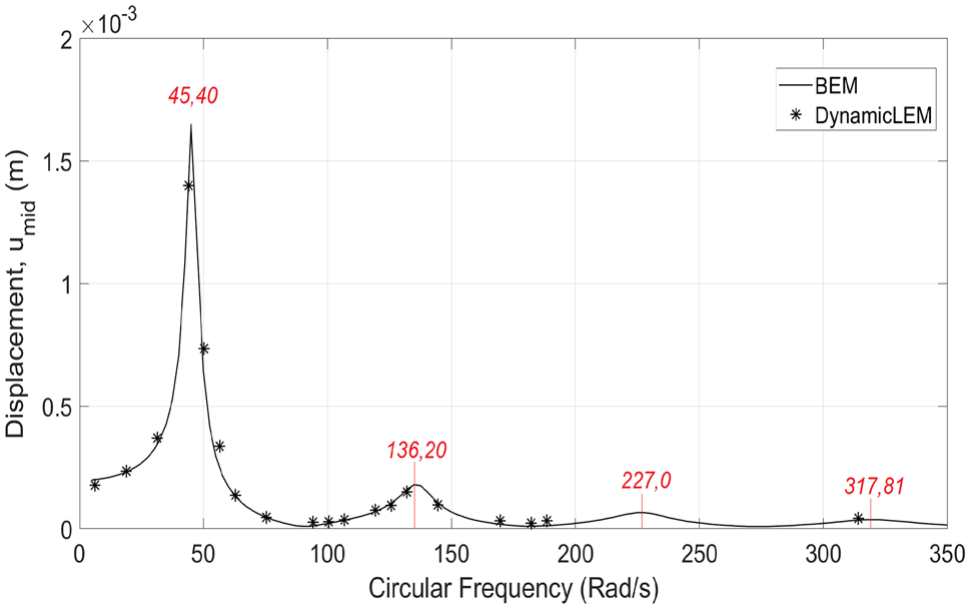
The comparison of BEM solution and dynamic lattice method results of displacements in mid-point of upper boundary ($u_{mid}$) under different circular frequencies.

With use of the dynamic lattice method, as described in “Implementation of the dynamic lattice method”, the problem is solved in the time domain using different harmonic $sin(\omega t)$ functions. The ordinary frequencies (*f*) shifted during the simulations from 1 to 50 Hz, where the angular frequencies are equal to $2\pi f$. The time step ($\Delta t$) is set to be $\Delta t=1.0e-05$, where the problem is solved for a duration of 1*s*. The similar material properties and boundary conditions are considered. The total number of generated elements using LEM is 10561, where the minimum wavelength ($\lambda$) at 50 Hz to element length (*l*) ratio is 21. For both the dynamic lattice method and BEM, the structural dynamic responses under different circular frequencies (*rad*/*s*) are illustrated in Fig. 6.

According to the recorded displacements in the mid-point of upper boundary ($u_{mid}$) vs. circular frequency results provided in Fig. 6, it can be concluded that the dynamicLEM solution provides accurate results, similar to the BEM solution as well as the analytical solution found in^50^. The results of the analytical solution of the resonance frequencies (45.50, 136.20, 227.0, 317.81 rad/s) obtained from the Eq. (20) are also marked on the figure. The major factor that affects the accuracy of the dynamicLEM solution is the wavelength to elements length ratio^51^. In the conducted parametric study, it’s found that when the wavelength to elements length ratio is kept higher than 10, the dynamicLEM solution provides accurate results.

## Simulation of wave fields in cracked and heterogeneous materials

For the analysis of wave fields in arbitrarily damaged and heterogeneous materials it is essential that the wave field is simulated considering all the wave field affecting perturbations. The following essential factors can be categorized for the modeling:

- Crack dependent criteria: length, thickness, orientation and location
- Domain dependent criteria: characteristics and properties, such as stiffness, anisotropy and heterogeneity factors
- Excitation dependent criteria: source, wavelength, frequency and magnitude
- Model dependent criteria: element length size and mesh irregularity

**Figure 7:**
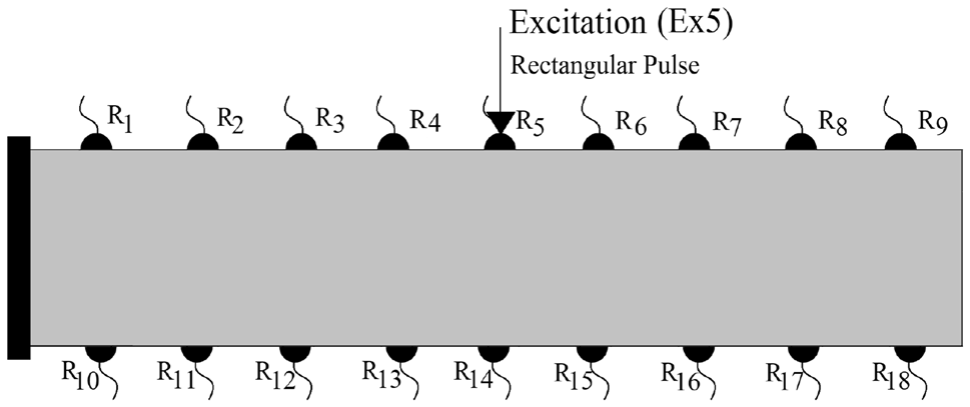
The defined excitation source and receiver sensors on a 2D cantilever beam.

In this study, we investigated how the following conditions effect the disturbance of displacement wave fields: single crack orientation, multiple discontinuities, particle heterogeneity similar to a concrete body, and randomly distributed mineral heterogeneity mimicking rock geomaterials. The Fig. 7 depicts the generated cantilever beam element with the receiver sensors located on the outer surfaces (boundaries). The dynamic excitation is also carried out through these predefined receiver (reference) sensors. The arrival of first and second displacement wavefronts can be measured in each receiver sensor, which can be accessed to detect the discontinuities^52^. In all of the simulated results, the considered frequency of a single rectangular pulse is 0,2 MHz. The Young’s modulus of a homogeneous medium is assumed to be equal to 3 GPa, where the Poisson’s ratio is equal to 0.2. The randomness factor of the generated mesh is 0.5. The mesh size is kept constant and equal to 800x160, where the total number of generated nodes and lattice elements are equal to 128000 and 382081, respectively. Therefore, the minimum ratio of wavelength to element length is kept as low as 22. The maximum equivalent diameter of polygonal Voronoi cells is equal to 0.125 mm and the damping ratio is assumed to be equal to zero. The applied magnitude of the excitation pulse is equal to 10 N, which is low enough to avoid any frack occurrence and propagation. Here, the Newton-Raphson Jacobian is implemented to solve the system of nonlinear equations with multiple variables as explained in “Implementation of the dynamic lattice method”. The convergence is granted when the maximum error between two subsequent iterations is lower than 1.0e-10. This error margin is mainly achieved after the third iteration. The non-linear dynamic frack simulation and its propagation is not in the scope of this research work.

The natural frequency of a simulated domain is dependent on the continuum effective stiffness and assigned masses on the Voronoi Cells. In each model setup, the same mesh with similar Voronoi masses is simulated. Therefore, the natural frequency of a system (cantilever beam) only varies with the assigned different effective stiffness values.

### Wave fields in the fractured materials

In order to investigate the disturbance of wave fields in fractured materials, a cantilever beam body as shown in Fig. 7 is considered. Initially, three homogeneous domains with different discontinuity conditions are simulated:

- NC - homogeneous beam element with no damage
- SC1 - homogeneous beam body with predefined single crack, where the crack orientation is perpendicular to the loading direction
- SC2 - homogeneous beam body with predefined single crack, where the crack orientation builds an angle of 45$^{\circ }$ with the horizon

**Figure 8:**
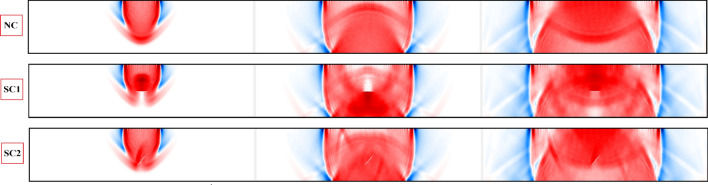
Plotted displacement wave fields in different time steps for each generated discontinuity condition: NC, SC1, and SC2.

**Figure 9:**
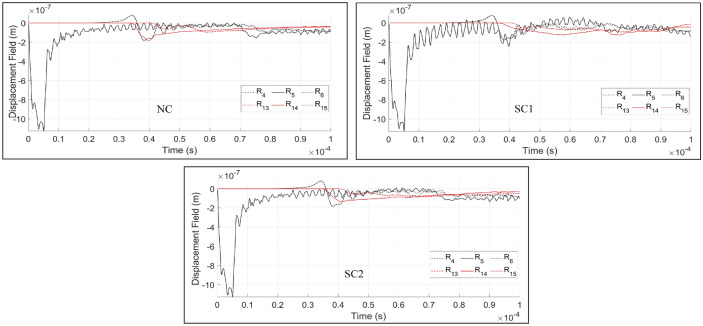
The recorded time histories at the receiver sensors under consideration of different crack conditions. Besides the direct wavefronts, the reflected and diffracted wave fronts are clearly visible by the different arrival times.

A single rectangular pulse is excited through receiver sensor R5, EX5. The simulated results according to dynamicLEM are provided in Figs. 8 and 9. Figure 8 illustrates the displacement wave field through the simulated beam body (qualitative results). In these results, the P-wave, SV-wave and Rayleigh surface waves are clearly visible and detectable. The time history of the displacements in the reference sensors ($R_4$, $R_5$, $R_6$, $R_{13}$, $R_{14}$, and $R_{15}$) are plotted in Fig. 9 (quantitative results). The disturbance of the wave fields due to the predefined discontinuities in SC1 and SC2 models are visible when compared to the NC model. Additionally, the arrival of the first and second wavefronts can be used to detect the location, orientation and length of the discontinuities in the domain. The wave field covers direct, diffracted and reflected waves around the crack within the domain. In these simulations, the time step ($\Delta t$) is equal to $\Delta t=1.0e-07$. The convergence of the dynamic solution depends on the size of the time steps.

**Figure 10:**
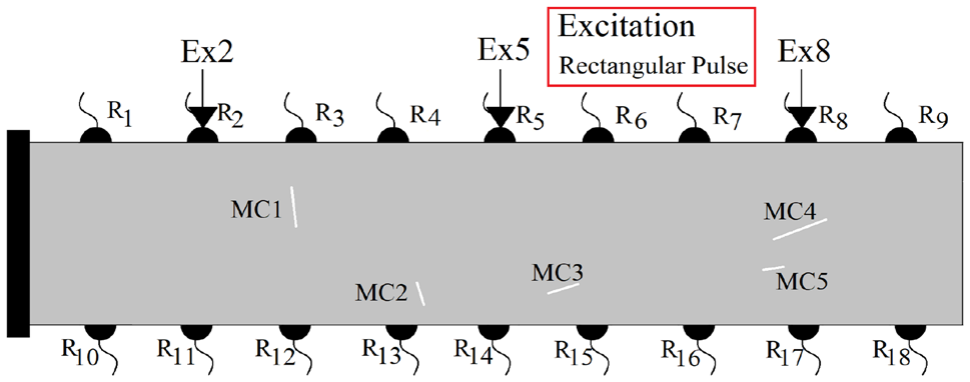
The generated random discontinuities and assigned excitation sources in $R_2$, $R_5$ and $R_8$.

**Figure 11:**
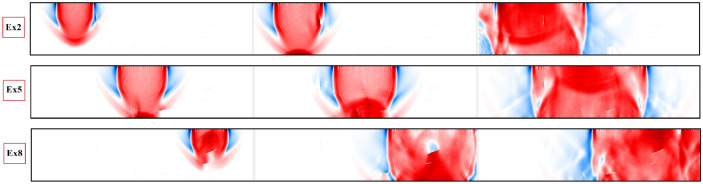
Plotted displacement wave fields in different time steps for each dynamic excitation condition: Ex2, Ex5, and Ex8. For better visualisation of the wave disturbance and dispersion different time steps are plotted.

**Figure 12:**
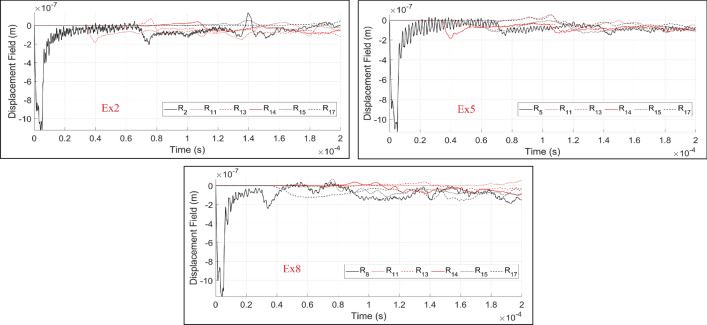
The recorded time histories at the receiver sensors ($R_{i}$) under consideration of different dynamic excitation sources: Ex2, Ex5 and Ex8.

In the next series of the results, multiple predefined discontinuities are generated inside the homogeneous beam element as shown in Fig. 10. The ability of the proposed dynamic element method to image even complex wave field patterns is investigated here. In the presented setup, five cracks (MC1:MC5) of different locations, lengths and orientations are randomly generated (Fig. 10). Three excitation positions, Ex2, Ex5 and Ex8 are used to excite the domain independently where the wave fields at the receivers can then be analyzed. Similar to the previous results, qualitative and quantitative results of the simulations are presented in Figs.11 and 12. Due to higher discontinuity of the simulated domain, a greater disturbance of wavefronts and the recorded time histories of the displacements are observed. For the identification of multiple discontinuities, analysis of the recorded time histories under multiple excitation sources are required. In the provided results, besides the excitation source, the recorded time history at $R_{11}$, $R_{13}$, $R_{14}$, $R_{15}$ and $R_{17}$ sensors are plotted.

### Wave fields in heterogeneous materials

**Figure 13:**
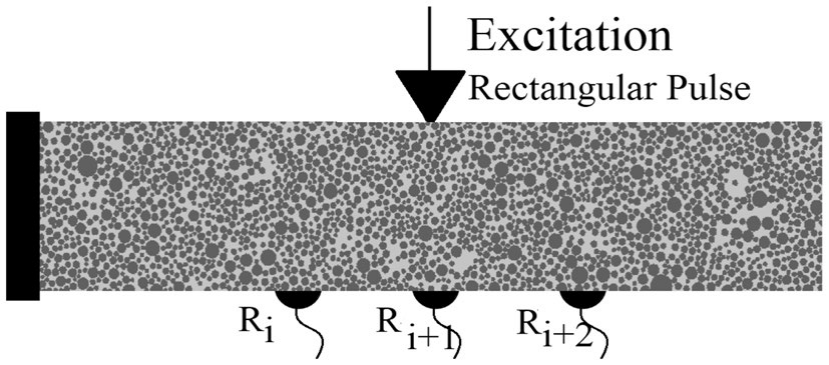
The generated concrete beam structure composed of aggregates and cement.

One of the main advantages of the lattice element method over conventional continuum methods is its ability to simulate the discontinuities while accounting for the inherent heterogeneity and irregularities in the particle scale. The irregularities such as the shape factors are already implemented while considering the irregular meshes and the defined randomness factor. In solid geomaterials, such as concrete or rock, the domain is composed of granular particles, cement (bond material) and the bond-particle interfaces as shown in Fig. 2. Two different heterogeneous domains, one similar to concrete and the other one similar to rock geomaterial, are simulated here to analyse and study the effect of the heterogeneity ratio on wave disturbance. As a result, not only the effect of the heterogeneity ratio on the wave disturbance can be investigated, but also the mineral cluster effect can be studied. Similar to concrete composition, a particle packing procedure is implemented here to generate a heterogeneous domain composed of two main components: aggregates and cement matrix. A rectangular beam element with non-uniform packing and the particles diameter varying from 0.5$\sim$4 mm is generated as shown in Fig. 13. The same boundary condition as previous setups is considered here and the rectangular pulse with an amplitude of 10 *N* is excited from the $R_5$ receiver. The stiffness of aggregates ($k_p$), bond cement ($k_b$) and aggregate-bond interface ($k_i$) are then assigned for each corresponding lattice element. Five different heterogeneity (stiffness) ratios are simulated:

- NC - $k_p=k_b=k_i$, where $E_i=3$ GPa
- CH1 - $k_p=2\times k_b=4\times k_i$, where $E_i=3$ GPa
- CH2 - $k_p=3\times k_b=30\times k_i$, where $E_i=3$ GPa
- CH3 - $k_p=5\times k_b=50\times k_i$, where $E_i=3$ GPa
- CH4 - $k_p=20\times k_b=200\times k_i$, where $E_i=3$ GPa

**Figure 14:**
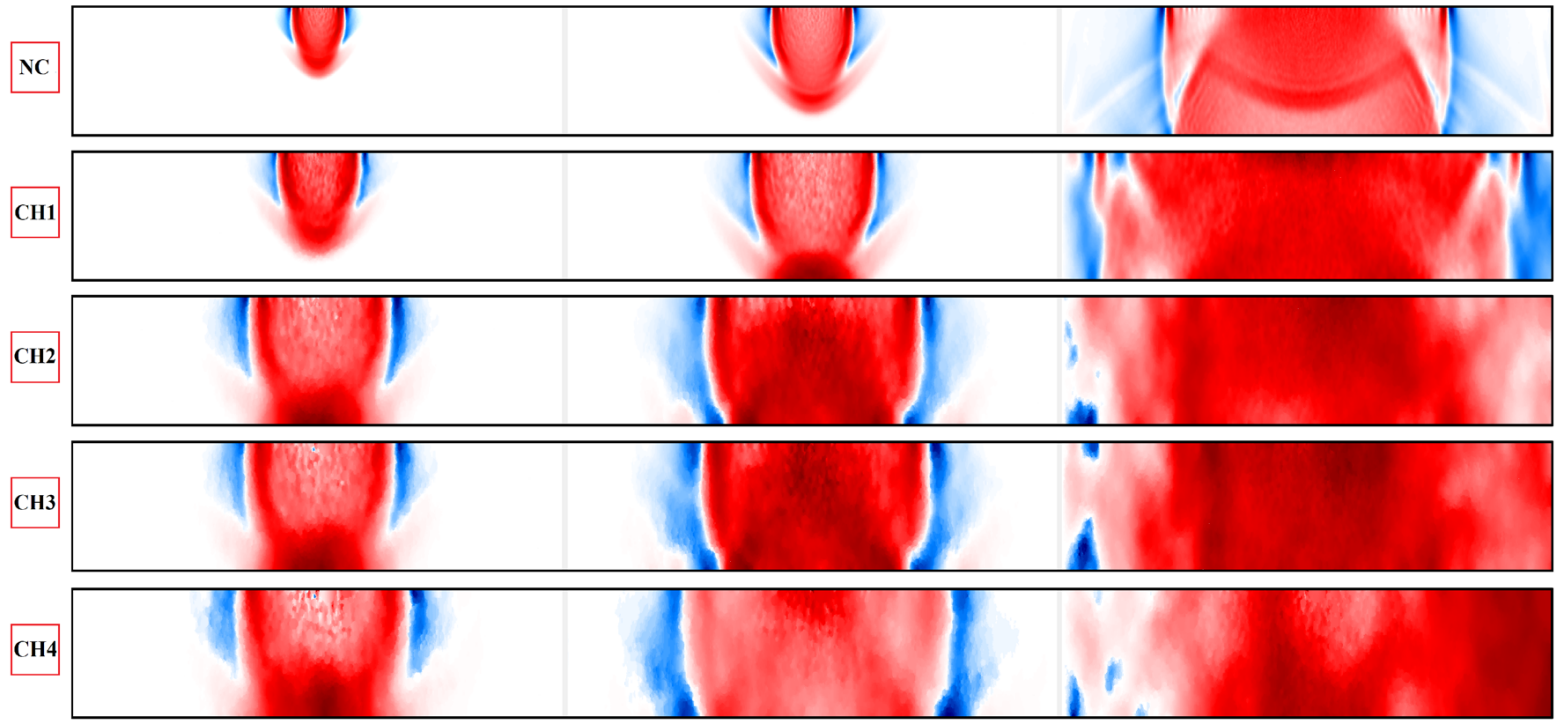
Plotted displacement wave fields in the same time steps (2, 3 and 10 $\times 10^{-5}$ (s)) and different heterogeneity ratios in concrete beam body: NC, CH1, CH2, CH3 and CH4.

**Figure 15:**
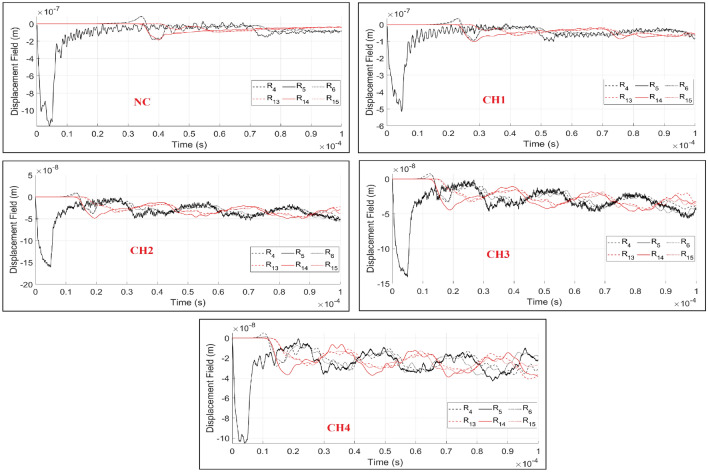
The recorded time histories at the receiver sensors ($R_{i}$) under consideration of different heterogeneity ratios in concrete beam domain: NC, CH1, CH2, CH3 and CH4.

The qualitative and quantitative results of DynamicLEM in a heterogeneous concrete body are presented in Fig. 14 and Fig. 15. Under the assumption of different heterogeneity ratios, the natural frequencies of the simulated domains are also affected. According to the results, the wave fields exhibit a larger diffusion and noise with a heterogeneity ratio increment in the domain. Due to the increased wave velocity in CH3 and CH4, the simulation time step is reduced to $\Delta t=1.0e-08$, however the frequency is kept constant and equal to 0.2 MHz. The value of the time step is dependent on domain size, elements size, frequency and material property. The assigned $\Delta t$ assures the convergence of the iteration method. The higher the difference between the stiffness of the mediums, the greater the magnitude of the reflected wave fronts. Visualization and investigation of particle scale heterogeneity behaviour by ordinary continuum-based methods are not possible, which opens a new research field to study these effects in detail by means of dynamic LEM.

**Figure 16:**
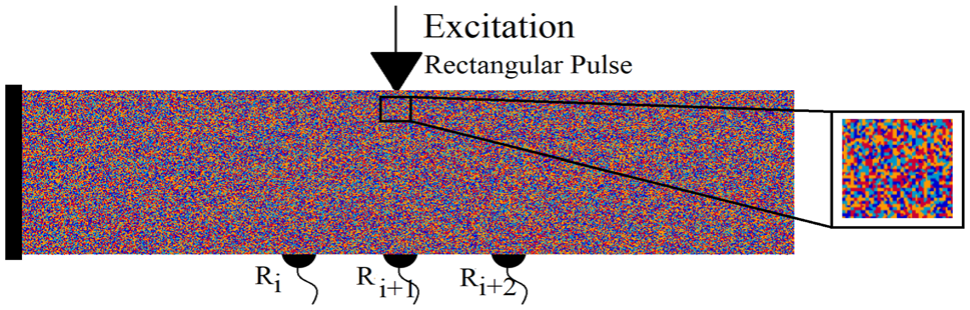
The randomly distributed heterogeneity inside the beam body to mimic a rock geomaterial.

As a follow up, qualitative and quantitative analyses of a wave field disturbance under multiple heterogeneity ratios are performed for a medium with randomly distributed heterogeneity. In this setup, a stochastic distribution of heterogeneity in each generated Voronoi cell is carried out. Therefore, instead of a cluster of cells representing a similar component as shown in the previous example, in this example, each Voronoi cell individually represents a unique mineral. The beam body without any predefined discontinuity is composed of four different minerals as shown with dark blue (DB), light blue (LB), red (R) and orange (O) cells in Fig. 16. The interface values between two minerals (i and j) are determined based on the equivalent value:

21 $$\begin{aligned} E_{int}=\frac{2\times E_i\times E_j}{E_i+E_j} \end{aligned}$$

Here, $E_{int}$ is the Young’s modulus of interface element between minerals *i* and *j*. This also highlights the importance of micro- and nanoscale THM properties in heterogeneous material, which requires further investigations.

In order to investigate the effect of a heterogeneity ratio on wave fields and to determine the critical heterogeneity ratio threshold, the following ratios are considered and simulated:

- NC - homogeneous domain, where $k_{DB}=k_{LB}=k_{R}=k_{O}$
- RH1 - heterogeneous domain with two distinct minerals, where $k_{DB}=k_{LB}=2k_{R}=2k_{O}$
- RH2 - heterogeneous domain with two distinct minerals, where $k_{DB}=k_{LB}=10k_{R}=10k_{O}$
- RH3 - heterogeneous domain with four distinct minerals, where $k_{DB}=2k_{LB}=3.4k_{R}=10k_{O}$
- RH4 - heterogeneous domain with two distinct minerals, where $k_{DB}=k_{LB}=30k_{R}=30k_{O}$
- RH5 - heterogeneous domain with two distinct minerals, where $k_{DB}=k_{LB}=50k_{R}=50k_{O}$
- RH6 - heterogeneous domain with two distinct minerals, where $k_{DB}=k_{LB}=80k_{R}=80k_{O}$
- RH7 - heterogeneous domain with four distinct minerals, where $k_{DB}=2k_{LB}=10k_{R}=100k_{O}$
- RH8 - heterogeneous domain with two distinct minerals, where $k_{DB}=k_{LB}=200k_{R}=200k_{O}$
- RH9 - heterogeneous domain with four distinct minerals, where $k_{DB}=2k_{LB}=40k_{R}=200k_{O}$

**Figure 17:**
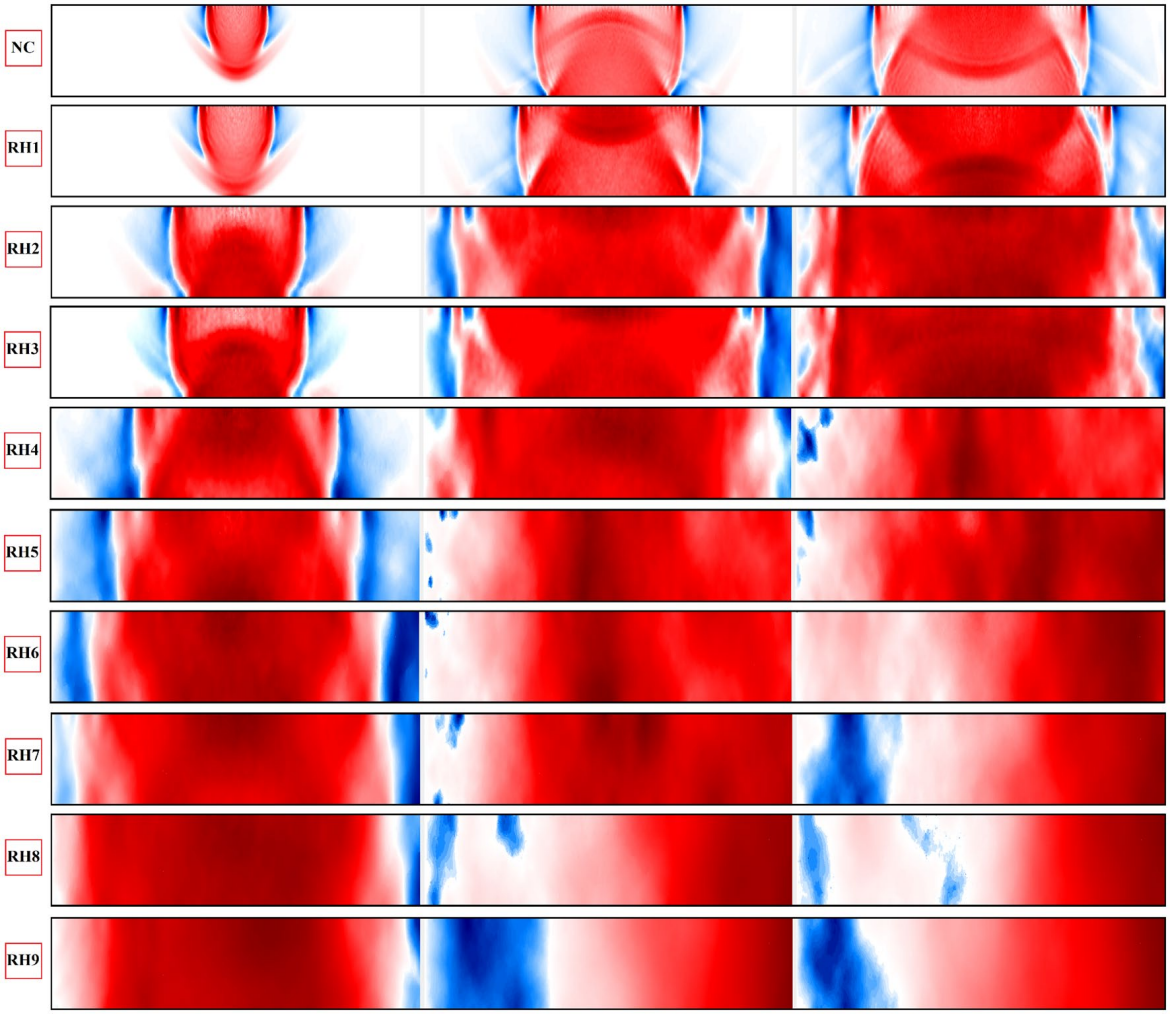
Plotted displacement wave fields in the same time steps (3, 7 and 10 $\times 10^{-5}$ (s)) and different heterogeneity ratios in the rock domain: NC, RH1:RH9.

**Figure 18:**
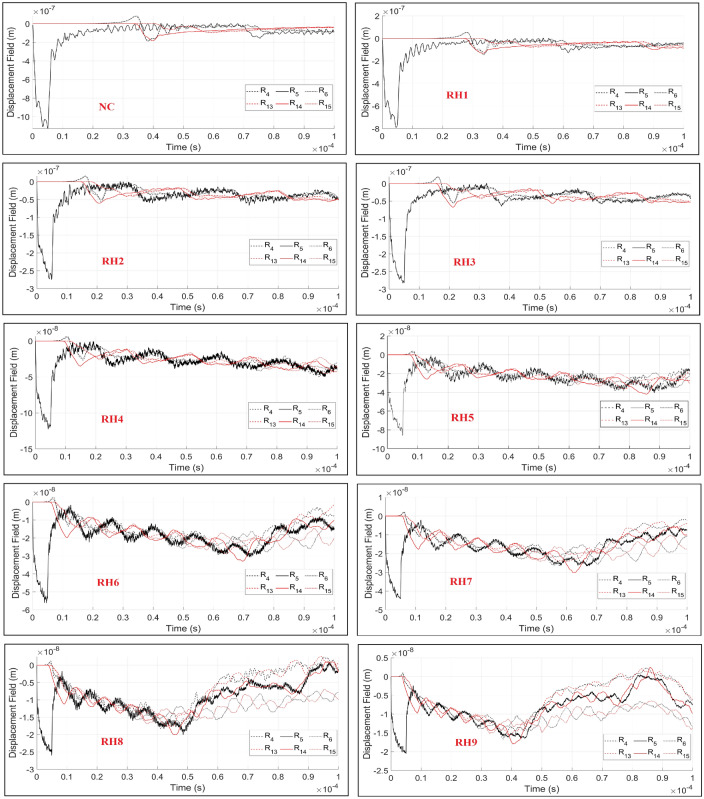
The recorded time histories at the receiver sensors ($R_{i}$) under consideration of different heterogeneity ratios in rock domain: NC, RH1:RH9.

Similar to the previous setup, a single rectangular pulse is excited through the R5 reference point (Ex5). With increasing the wave velocities, the time step is decreased to grant the convergence of the numerical solution. Therefore, the simulation time step is reduced to $\Delta t=1.0e-08$, where the frequency is kept constant and equal to 0.2 MHz. The quantitative and qualitative recorded displacement time histories in all of the simulation setups are presented in Figs. 17 and 18. In all of the simulations a similar setup with same mesh size (same masses), randomness factor and stochastic heterogeneity is considered. The adapted effective stiffnesses of domains results in different natural frequencies in each setup. The simulated damping ratio is equal to zero, which grants continuous vibration of the beam structure.

Besides the simulation of wave propagation under isotropic conditions, the dynamic LEM is able to simulate wave vibrations in an anisotropic media. Based on the presented results for both concrete and rock type geomaterials, it can be concluded that:

- Increasing the stiffness heterogeneity between particle, bond matrix and aggregate-cement interface induces excessive disruption on the wave fronts. The magnitude of the reflected wave fronts is increased when the ratio of the stiffness is enlarged. The recorded and plotted data are dependent on the natural frequency of a beam structure. In all of the setups presented for a concrete body, the assigned masses (generated mesh) on Voronoi cells are constant. With increasing the stiffness of a domain, the wave velocity also becomes greater. Although the increment of the heterogeneity induced wave dispersion in the concrete body, the evaluation of the outcomes based on the arrival of P and SV waves is still possible.
- In a rock domain, the effect of heterogeneity ratios as well as number of minerals on wave disturbance is analysed. Similar to the concrete body, increasing the stiffness heterogeneity induces excessive disruption on the wave fronts. In contrast to the concrete body, after a certain stiffness heterogeneity ratio, the evaluation of the outcomes based on the arrival of P and SV waves is not possible. This is clearly visible from the plotted time histories, starting from RH5, where at around $0.9\times 10^{-4}$ the accumulated noises disrupt the wave fronts.
- The total number of minerals in a rock domain has an effect on the disturbance of the wave fronts. This is clearly distinguishable when comparing the RH8 and RH9 results, where the maximum stiffness heterogeneity ratio is equal to 200. In RH8, the disturbance of wave fronts starts from $0.45\times 10^{-4}$, where in RH9, the wave dispersion begins at $0.4\times 10^{-4}$. It should be noted that the natural frequencies of these domains are not equal, which can also induce different vibration responses. The results clearly depict that increasing the number of minerals induces larger disturbance on the wavefronts.
- While comparing the rock and concrete bodies it is clear that in a heterogeneous rock body of a similar stiffness heterogeneity ratio, the wave dispersion and scattering is higher. In contrast to CH4, in RH9, where in both setups the maximum stiffness heterogeneity ratio is equal to 200, the evaluation of the arrival of P and SV waves is not possible. This again emphasises the importance of considering the inherent heterogeneity in numerical simulations. Identification of discontinuities based on arrival of first and second P and S waves will then be a challenging task, which requires further investigations.
- A small stiffness transition zone (mineral to mineral) produces a larger variation of P and S wave velocities, which leads to larger reflections and dispersion of waves. Increasing the thickness of the transition zone (cluster of minerals) results in gradual variation of seismic velocities. This also reduces the energy of reflected and diffracted waves, which eventually has less impact on the disturbance of wave fronts.

## Discussion and conclusion

In this study, we investigated the application of a 2D dynamic lattice in the simulation of displacement wave fields in a fracked and heterogeneous medium. The proposed dynamic lattice model based on the equation of motion of forced vibration is not only used to simulate the displacement wave fields in homogeneous domain but also to illustrate qualitatively and quantitatively the disturbance of wave fields in the discontinuous and heterogeneous medium. It is also possible to detect the defects using the dynamic lattice model, in which a high-frequency wave can be generated and recorded in the reference sensors. The dynamic lattice is also capable of detecting the location of the initiated crack in a stressed structure. In order to validate the in-house developed 2D dynamic lattice, the displacement vs. angular frequency results of a 2D plain strain domain are compared to the boundary element method (BEM) solution. It is shown that with larger wavelength to element length ratios, the lattice results provide great accuracy.

The advantage of dynamic lattice in the displacement wave field simulation lies in its embedded irregularity in domain discretization, its ability to define discontinuities and particle heterogeneity, simulate frack initiation and propagation as well as the stress redistribution and concentration upon crack propagation. Similar to discrete methods, the lattice method also considers the inherent heterogeneity in particle scale. To better illustrate this advantage, the developed dynamicLEM is used to simulate the displacement wave fields in heterogeneous concrete and rock geomaterials. The results indicate that with increasing the maximum heterogeneity ratio (stiffness ratio), the disturbance of wave fields becomes greater. After a certain heterogeneity threshold, the quantitative evaluation of dynamic results with conventional methods is not possible. It is also shown that increasing the number of mineral compositions in a rock body has a considerable effect on the wave field disturbance. Comparing the rock and concrete beam bodies indicates the importance of mineral distribution scheme and particle sizes on dispersion of wavefronts. It is shown that in a same stiffness heterogeneity ratio, the reflection and diffraction of waves in a rock domain (individual mineral distribution) are stronger than a concrete domain, where a cluster of Voronoi cells represent an individual aggregate. The results cohere with the theory, where a small stiffness transition zone (mineral to mineral) produces a larger variation of P and SV wave velocities compared to a larger transition zone (cluster of minerals). The simulations are performed on a Desktop-PC with a Xeon processor (2.10 GHz) with a total number of 16 cores, and the computational time for a single simulation is approximately 24 hours. In the developed explicit algorithm, parallel computing is partially implemented, however the main solver is dependent on a performance of a single core. For larger domain simulations, parallelization and optimization of the developed algorithm is essential.

The dynamic lattice element is capable of modeling the wave field’s disturbance and dispersion when a discontinuity and heterogeneity in the domain is present. The identification of discontinuities in heterogeneous materials is a challenging task, where particle scale models similar to dynamicLEM can provide accurate outcomes. However, the simulation of large domains with high frequencies requires a substantial number of elements, not only to model the particle size effect but also to reach greater wavelength to elements length ratio and avoid numerical difficulties. One way to overcome this obstacle is to consider the artificial neural networks (ANN) models to predict the location of the discontinuities in the homogeneous and heterogeneous bodies^40^. The recorded displacement wave fields at each reference point in dynamicLEM are considered as training data for developing an ANN method to not only decrease the computational costs but also increase the accuracy of the crack predictions. Eventually, for any given wave spectrum that can be obtained from the field applications, the ANN method is able to predict the location, length and orientation of the existing discontinuities.
